# Sweeteners and the Gut Microbiome: Effects on Gastrointestinal Cancers

**DOI:** 10.3390/nu15173675

**Published:** 2023-08-22

**Authors:** Raghad Khalid AL-Ishaq, Peter Kubatka, Dietrich Büsselberg

**Affiliations:** 1 Department of Physiology and Biophysics, Weill Cornell Medicine-Qatar, Education City, Qatar Foundation, Doha 24144, Qatar; rkmalishaq@hotmail.com; 2 Department of Medical Biology, Jessenius Faculty of Medicine, Comenius University in Bratislava, 036 01 Martin, Slovakia; peter.kubatka@uniba.sk

**Keywords:** sweeteners, non-nutritive sweeteners, steviol, saccharin, gut microbiome

## Abstract

Worldwide, the demand for natural and synthetic sweeteners in the food industry as an alternative to refined sugar is increasing. This has prompted more research to be conducted to estimate its safety and effects on health. The gut microbiome is critical in metabolizing selected sweeteners which might affect overall health. Recently, more studies have evaluated the relationship between sweeteners and the gut microbiome. This review summarizes the current knowledge regarding the role played by the gut microbiome in metabolizing selected sweeteners. It also addresses the influence of the five selected sweeteners and their metabolites on GI cancer-related pathways. Overall, the observed positive effects of sweetener consumption on GI cancer pathways, such as apoptosis and cell cycle arrest, require further investigation in order to understand the underlying mechanism.

## 1. Introduction

### 1.1. Natural and Synthetic Sweeteners

Worldwide, the consumption of sugars of glucose or fructose syrup and sucrose has dramatically increased [1,2]. This prompted scientific discussions about their diverse effects on health conditions such as obesity, inflammatory responses, and metabolic conditions, which has motivated people to use natural and zero-caloric sweeteners as alternatives [3]. Sweeteners substitute for sugar, as they mimic their sweet taste [4]. Non-nutritive sweeteners (NNS) are often used to substitute refined sugar in the food industry and the human diet since they provide the desired sweet taste while having a lower calorie content [5,6]. They can be naturally or synthetically produced, with the former having a higher quality, sweetness intensity, and quantity [7]. The consumption of NNS is not limited to people with metabolic conditions such as diabetes, but also to the general population, as they are commonly found in various food products [8]. As a result, efforts are made to measure and ensure the safety of those products. For example, the US Food and Drug Administration (US FDA) follows a specific process to approve new sweeteners, which includes determining the appropriate intake amounts, estimating toxicity levels, and evaluating the cumulative effects of the sweeteners. However, the recent WHO statement that lists aspartame as a potential carcinogen shows the necessity of investigating the mechanistic effects of those sweeteners on health and how they are related to cancer [2,9]. Examples of approved natural and synthetic sweeteners include steviol glycoside and saccharin, respectively [10].

### 1.2. Metabolization of Sweeteners by Gut Microbiome

Recent findings have linked the gut microbiome to multiple health implications, from diabetes to colorectal cancer [11]. The gastrointestinal tract is inhabited by prevalent microbial species such as bacteria, viruses, and fungi [12]. Those communities play an important role in the host’s metabolism, growth, and immunity [13]. Diet is an important factor that modulates the gut flora’s composition, metabolism, and function [14]. Global interest in NNS products metabolized by the gut microbiome and their potential biological effects has increased recently [15]. Biological effects such as the administration of acesulfame K-depleted *Akkermansia muciniphilia*, which was associated with increased glucose intolerance, have been observed [16]. Additionally, administering 0.3 mg/kg of neotame in mice reduced the abundance of *Firmicutes* while enhancing the abundance of *Bacteroidetes* [17]. Collectively, these results suggest an effect of NNS on the gut microbiome and an impact on the host’s physiological status. Possible mechanisms of interactions may be: (i) interactions between NNS and taste receptors with high affinity to gut microbes; and (ii) NNS acting directly on the gut barrier [18,19]. More efforts are required in order to support those possible mechanistic interactions.

### 1.3. Sweeteners and Gastrointetional Cancers

Linking the use of sweeteners in the food industry as alternatives to sugar and the safety concerns associated with their use is widely debated, with conflicting findings regarding their role in disease etiology [20]. Various research studies have investigated the potential risk of using sweeteners for gastrointestinal cancer [21]. A meta-analysis reviewed the data obtained from eight studies on 1,043,496 individuals, among whom some were diagnosed with different types of GI cancers (3271 pancreatic, 304 esophageal, 395 gastric, 3008 colorectal, and 598 oropharyngeal). The analysis results indicated a 19% reduction in the risk of developing luminal GI cancer after consuming sweeteners [22]. Additionally, a study of 1010 participants from Italy with different types of GI cancers reported an absence of effects on GI cancer development when using commonly available sweeteners [23].

Moreover, in another meta-analysis study that evaluated 25 observational studies, the reported results supported the lack of a link between overall cancer incidence and mortality with the consumption of artificial sweeteners [24]. Despite that, recent findings have reported an association between sweetener intake and the risk of cancer development. In a large French cohort, the consumption of sweeteners, especially aspartame and acesulfame-K, was associated with the risk of cancers according to the Cox proportional hazards models which they followed [25]. Those controversial results indicate the urgent need for unified efforts to standardize protocols, develop statistical methods, and reduce confounding results and biases to advance the field further and re-evaluate food additives’ safety and quality, as this issue greatly affects people’s life and health.

The literature discusses the role of the gut microbiome in metabolizing selected sweeteners and their influence on GI cancer development. Here, we evaluate and analyze published studies that report the influence of bacterial species on both natural and artificial sweeteners (steviol glycoside, glycyrrhizin, neohesperidine dihydrochalcone, saccharin, and sucralose). Furthermore, we assess the impact of the selected sweeteners alone, or, if supported by the literature, their metabolites, in cancer-related pathways. Finally, we identify gaps in the current research.

## 2. Search Strategy and Selection Criteria

Medline, Scopus, and PubMed were searched for manuscripts published from 2000 to 2023 using the search terms “GI cancers”, “microbiota”, “sweeteners”, “microbiome profile AND sweeteners”, “gut microbiota enzymes”, “Steviol glycoside AND GI cancers”, “Glycyrrhizin AND GI cancers”, “Neohesperidine dihydrochalcone AND GI cancers”, “Saccharin AND GI cancers”, and “Sucralose AND GI cancers”. The search yielded a total of 400 articles. We selected 104 articles and analyzed them in detail for this review. Eligible studies included in vivo, in vitro, and clinical trial publications addressing the metabolisms of selected bacteria on sweeteners and their role in the development and complications of gastrointestinal cancers. Sweeteners that did not address/report such metabolisms were excluded. Also, the effects of sweeteners on other cancer types were excluded.

## 3. Sweeteners and the Gut Microbiome

Throughout our research, the relationships between three natural sweeteners (steviol glycoside, glycyrrhizin, and neohesperidine dihydrochalcone) and two synthetic sweeteners (saccharin and sucralose) and the gut microbiome have been discussed. Here, we discuss the results and examine these relations in detail in order to provide insight into the mechanisms and metabolization of these sweeteners.

### 3.1. Steviol Glycoside

*Stevia rebaudiana* is a shrub found mainly in South America, specifically in Brazil and Paraguay [26]. It belongs to the family *Asteraceae*, and it is used as a natural and non-caloric sweetener because of its high sweetness intensity, which is caused by steviol glycosides [27]. Purified steviol glycoside extracts have been used in the food industry as sweeteners in many regions [28]. The European Food Safety Authority (EFSA) thus reported the acceptable daily intake of steviol glycosides to be 4 mg/kg/day [29,30]. The leaves of *Stevia rebaudiana* contain several diterpene glycosides, such as rubusoside and steviolbioside [31]. Multiple in vitro studies have supported the metabolization of stevia extracts by the gut microbiome [32]. *Bacteroides* species in the gut play an important role in metabolizing two of the main components of *Stevia rebaudiana* by hydrolyzing rebaudioside A and stevioside to steviol in the gut [33]. This suggests that neither component is absorbed in the upper gastrointestinal tract [34]. Using the portal vein, the absorbed steviol reaches the liver for further metabolism to steviol glucuronide and is excreted in the urine [35].

### 3.2. Glycyrrhizin

One of the 300 active licorice compounds is glycyrrhizin, a triterpene saponin glycoside [36]. It is used as an herbal product in medicine due to its anticancer and anti-inflammatory activities [37]. It has a high sweetness intensity (up to 200 times sweeter than sucrose) [38]. Ingestion of less than 100 mg/day of glycyrrhizin is considered safe [39]. Due to its poor oral bioavailability, glycyrrhizin is metabolized by the gut microbiome [40]. Both *Eubacterium* and *Bacteroides* species are involved in the de-glycosylation of glycyrrhizin to a major product, glycyrrhizic acid, and a minor product, 18β-glycyrrhetic acid 3-O-monoglucuronide [41]. After that, both products reach the liver for further conjugation and reduction [42]. Both biliary and urinary excretions occur to the major parts of the products, respectively [43].

### 3.3. Neohesperidin Dihydrochalcone

**Figure 1:**
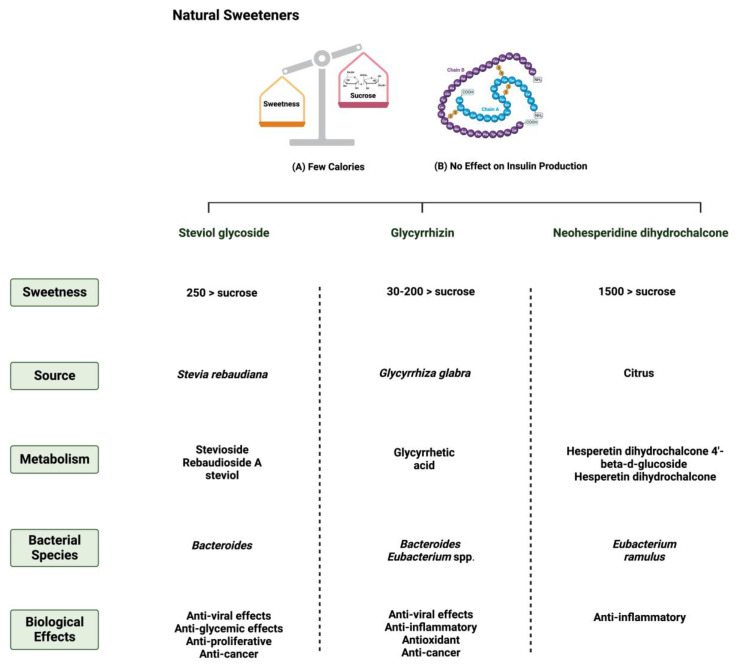
Overview illustration of natural sweeteners. The figure describes two of the main features of natural sweeteners. It also shows the sweetness intensity, the source of natural sweeteners, their metabolism by the gut microbiome, and their main biological effects. Created with BioRender.com (accessed on 15 July 2023).

**Figure 2:**
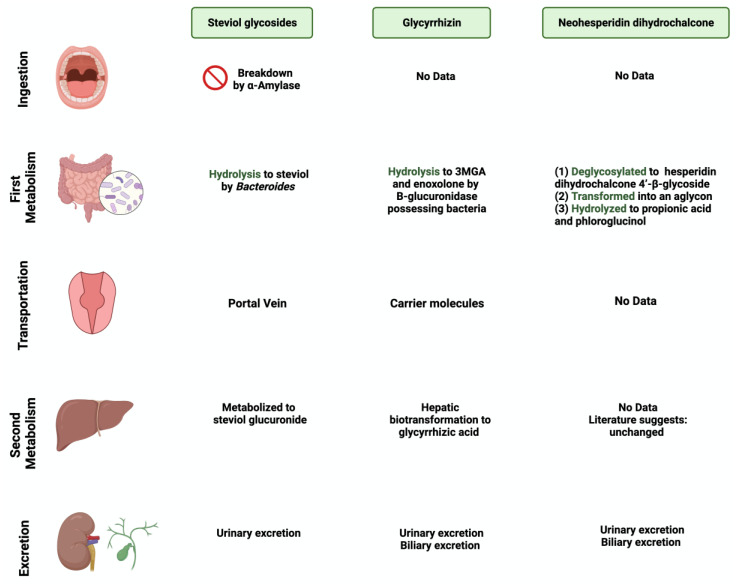
Overview illustration of natural sweetener consumption and metabolism. The figure is divided into different sites of metabolism for each of the natural sweeteners. Created with BioRender.com (accessed on 15 July 2023).

Neohesperidin dihydrochalcone (NHDC) is a natural sweetener found mainly in the skin of citrus fruits; it possesses high stability and solubility [44]. It is obtained and processed from its parent flavanone, neohesperidin, and has a sweetness intensity 250–1800 times higher than sucrose [45]. Despite that, the usage of NHDC as a replacement for sucrose is limited in the food industry due to its flavor formulation, texture, and size [46]. Although not widely known, the metabolism of NHDC by the gut microbiome has been discussed in the literature [47]. The metabolism starts with NHDC being deglycosylated to hesperidin dihydrochalcone 4′-β-glycoside, transforming into an aglycone. The final step of NHDC metabolism is the hydrolysis of the aglycone to propionic acid and phloroglucinol [48]. The products are then excreted either through urine or bile [49]. Figure 1 and Figure 2 summarize and provide an overview of the three natural sweeteners and their metabolism by the gut microbiome.

### 3.4. Saccharin

Saccharin (1,1-dioxo-1,2-benzothiazol-3-one), also known as E954, is a non-caloric sweetener used widely in the food industry [50]. It is found either in an acid form or bound to calcium or sodium (higher stability and solubility) [51]. Saccharin’s sweetness intensity is 300 times higher than sucrose [52]. The FDA considers saccharin consumption to be safe due to its inability to be metabolized by the body [53]. Once consumed, most of the ingested saccharin (85–95%) is absorbed and bound reversibly to plasma proteins when excreted in the urine. The rest passes through the GI tract to be eliminated, unchanged, in the feces [34]. Due to this, studies have investigated the influence of saccharin on gut microbiome composition. The administration of 90 mg of saccharin in rats did not alter the total number of anaerobic bacteria, but eliminated specific anaerobic groups in the cecal contents [54].

Additionally, rats receiving a 2.5% dose of saccharin inhibited the growth of three *Escherichia coli* strains and three *Lactobacillus* species [55]. These studies may suggest that even if the body does not metabolize the sweeteners, their consumption impacts the gut microbiome’s composition and function, which might alter the host’s health status. However, recent studies using advanced technologies are required in order to assess saccharin’s safety and effectiveness and to address the controversial results in the literature.

### 3.5. Sucralose

Sucralose, or E-955, is a low-caloric, non-nutritive synthetic sweetener and is very similar in structure to sucrose [56]. However, sucralose is formed when the three hydroxyl groups attached to the sucrose molecule are replaced by chlorine atoms [57]. It is 600 times sweeter than sucrose [58]. Like saccharin, sucralose is not metabolized by the body; however, unlike saccharin, most ingested sucralose passes through the GI tract to be eliminated in the feces. The rest reaches the kidneys for urinary excretion [59]. The administration of sucralose influences its abundance in the gut microbiome. The relative abundance of *Clostridium* cluster XIVa was affected in mice given 15 mg of sucralose/kg [9].

**Figure 3:**
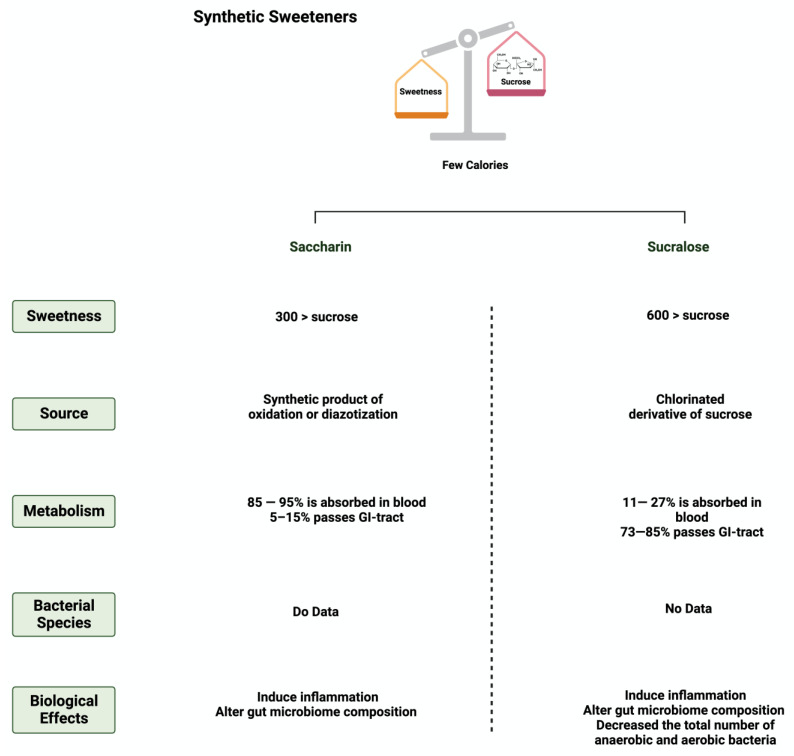
Overview illustration of synthetic sweeteners. The figure describes the main features of synthetic sweeteners. It also shows the sweetness intensities, the sources of the synthetic sweeteners, their metabolism by the gut microbiome, and their main biological effects. Created with BioRender.com (accessed on 15 July 2023).

**Figure 4:**
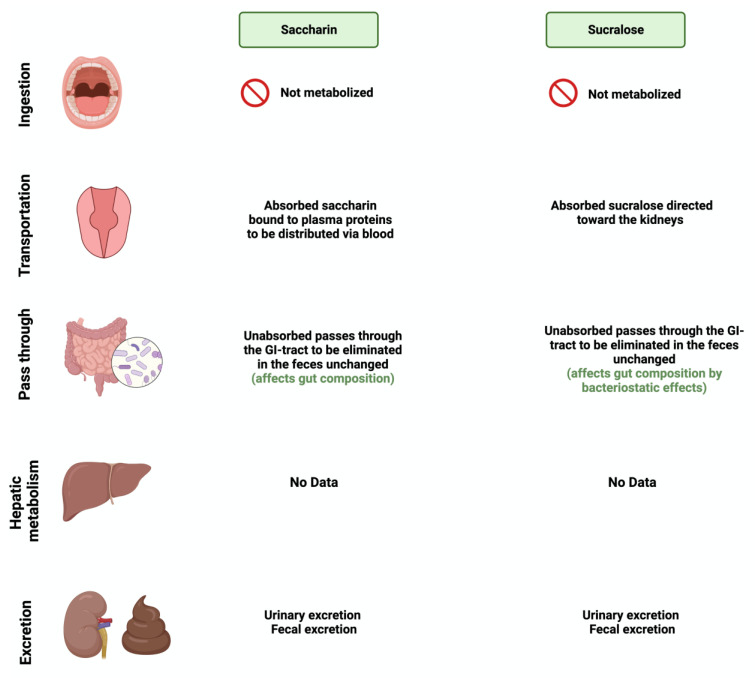
Overview illustration of synthetic sweetener consumption and metabolism. The figure is divided into different sites of metabolism for each of the synthetic sweeteners. Created with BioRender.com (accessed on 15 July 2023).

Additionally, sucralose administration for six months influenced the abundance of 14 different taxonomic levels, as well as the regulation of amino acids and chronic inflammation, in C57BL/6 mice [60]. This shows the urgent need for further research to investigate the observed effects on humans. Figure 3 and Figure 4 summarize and provide an overview of the two synthetic sweeteners and their metabolism by the gut microbiome.

## 4. Sweeteners’ Role in Gastrointestinal Cancers

The effect of natural and synthetic sweeteners on the development of organ-specific cancer has been discussed for years [61]. With the continued rise in the consumption rate of sweeteners worldwide, several reports have supported the positive influence of sweeteners on the development and progression of GI cancer [62]. Here, we will discuss the effects of the five sweeteners and, if available and supported by the literature, their metabolites on the major pathways impaired in GI cancers (apoptosis, NF-KB, and cellular arrest).

### 4.1. Apoptosis

**Figure 5:**
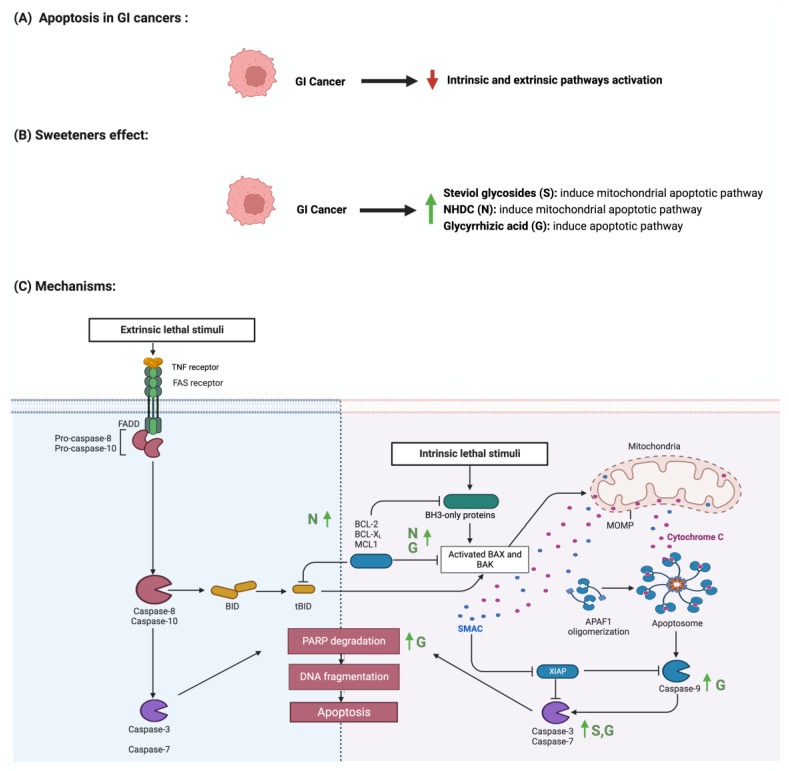
Illustrations of the influence of sweeteners on the apoptotic pathway in GI cancers. The figure highlights the pathological changes in apoptosis due to GI cancer, the sweeteners’ effects, and the mechanisms through which the sweeteners target the pathway. Created with BioRender.com (accessed on 15 July 2023).

Apoptosis is programmed cell death characterized by morphological and biochemical changes [63]. Its involvement in various processes, such as immune system development, makes it an essential physiological process [64]. When unregulated, it plays a role in the development of several diseases, such as autoimmune diseases, neurodegenerative disorders, and cancers [65]. Sweeteners have been reported to influence the process of apoptosis in cancers [66]. Steviol, a colonic metabolite, inhibits apoptosis in GI cancer cells as effectively as 5-fluorouracil (100 ug/mL) through the mitochondrial apoptotic pathway [67]. Additionally, in one study, steviol administration at a 1000 ug/mL concentration effectively reduced cell viability and induced apoptosis in colon cancer cells [68,69]. The results of a study that investigated the effect of 17 steviol derivatives on different cancer cell lines showed a potent cytotoxic effect of those derivatives on the cell lines [70]. Glycyrrhizin is also reported to possess apoptotic activities on GI cancers [71]. The administration of glycyrrhizin on SW48 colorectal cancer cells induced apoptosis as the levels of regulator proteins such as Bax expression increased and Bcl-2 levels decreased [72]. HT-29 colon cancer cells treated with different concentrations of *glycyrrhiza glabra* L. reported the induction of apoptosis at a concentration of 200 μg/mL [73,74]. Additionally, Wister rats administered 15 mg/kg of glycyrrhizic acid were reported to induce apoptosis, suppress precancerous lesion development, and reduce inflammation [75]. In a different study, the oral administration of glycyrrhizic acid (15 mg/kg) in Wister rats once a week for 15 weeks induced apoptosis by enhancing the expression of cleaved caspase 3 [76]. The induction of apoptosis through pro-caspases 3, 8, and 9 was reported in gastric cells treated with glycyrrhizic acid [77]. The sweetener neohesperidin dihydrochalcone, administered to an APC min/+ transgenic mouse model, inhibited colorectal tumorigenesis and induced apoptosis [78]. Phloroglucinol (PG), a metabolite of NHDC, induced apoptosis in HT-29 cells via overexpressed caspase-3 and caspase-8, modified Bcl-2 family proteins, and cytochrome c release [79]. In another study, PG protected mice’s intestinal damage from ionizing radiation by increasing apoptosis by affecting the p53, Bax, Bak, Bcl-2, and Bcl-X_S/L_ proteins [80] The literature still lacks the evidence to show the underlying mechanism of the observed effect of sweeteners on GI cancers. Figure 5 summarizes the effect of sweeteners on the apoptotic pathway.

### 4.2. The Nuclear Factor-κB Pathway

**Figure 6:**
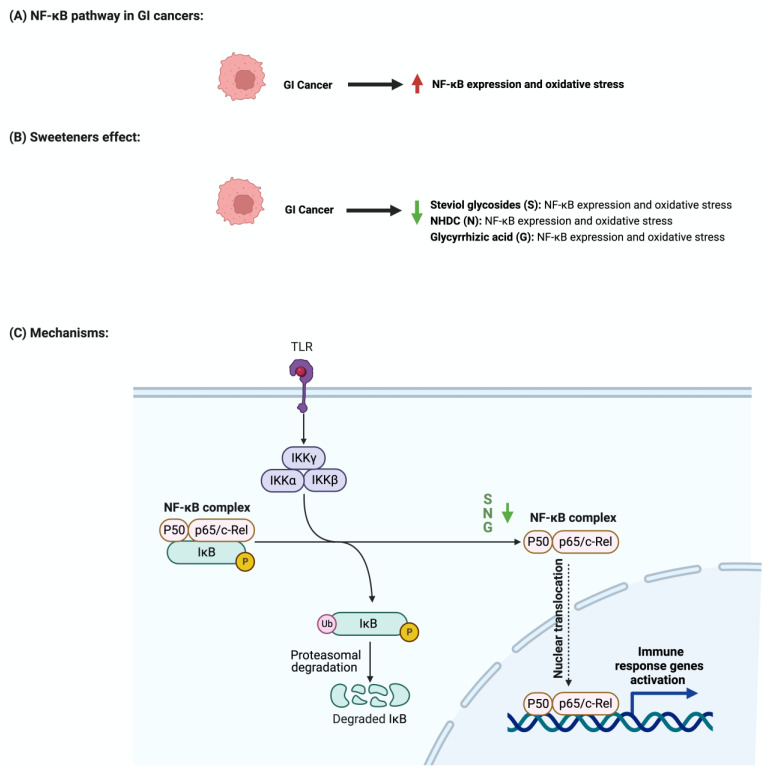
Illustrations of the influence of sweeteners on NF-κB in GI cancers. The figure highlights the pathological changes in NF-κB due to GI cancer, the sweeteners’ effects, and the mechanisms through which the sweeteners target the pathway. Created with BioRender.com (accessed on 15 July 2023).

The nuclear factor-κB (NF-κB) pathway regulates genes that regulate inflammatory and immune responses [81]. In cancer, NF-κB promotes cellular proliferation and metastasis and suppresses apoptosis [82]. Although not abundantly discussed in the literature, multiple reports support the role of sweeteners in NF-κB pathway regulation [83]. Stevioside administration to a colon carcinoma cell line (Caco-2) suppressed the expression of inflammatory cytokines IL-6, TNF-a, and NF-κB [84]. Additionally, the administration of glycyrrhizic acid inhibited NF-κB expression, which led to the deactivation of inflammatory mediators in colon cells [74,85]. In Wister rats, the administration of 15 mg/kg of oral glycyrrhizic acid reduced the expression of NF-κB, nitric oxide synthase (iNOS), and cyclooxygenase-2 (COX-2) [76]. Neohesperidin dihydrochalcone, along with the two other sweeteners, influenced NF-κB expression. Oral administration of neohesperidin dihydrochalcone in mice for six days attenuated the expression of NF-κB [86]. Neohesperidin dihydrochalcone inhibited the induced NF-κB expression in paraquat-induced acute liver injury [87]. More efforts and standardized steps are required in order to conduct more research in this field and to understand the underlying mechanism of this effect. Figure 6 summarizes the effects of sweeteners on NF-κB expression.

### 4.3. Cellular Cycle Arrest

**Figure 7:**
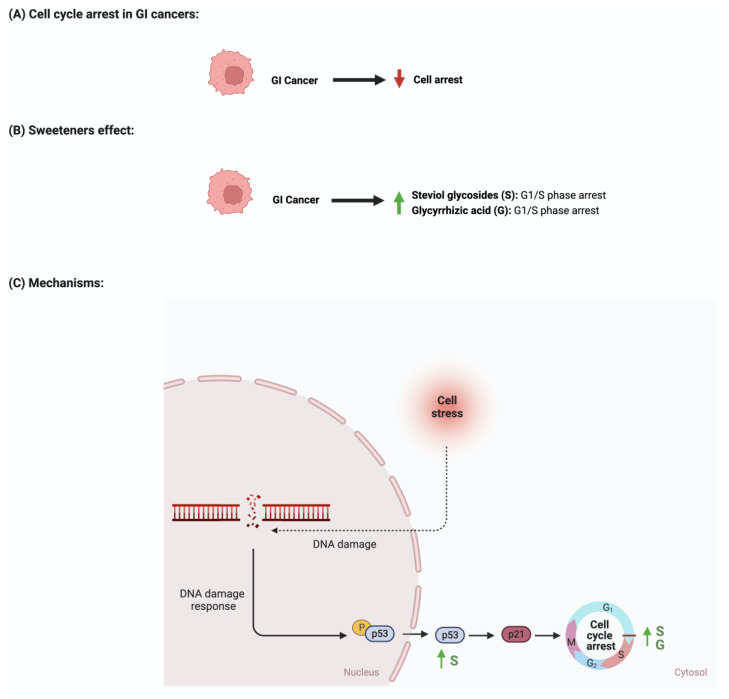
Illustrations of the influence of sweeteners on cell cycle arrest in GI cancers. The figure highlights the pathological changes in cell cycle arrest due to GI cancer, the sweeteners’ effects, and the mechanisms through which the sweeteners target the pathway. Created with BioRender.com (accessed on 15 July 2023).

The development and function of every tissue depend on the cellular decision to transition from a proliferative to an arrested state [88]. Cancerous cells dysregulate cell cycle arrest and continue to undergo uncontrolled cellular growth [89]. The effect of sweeteners on cellular cycle arrest is scarcely reported in the literature. In a study that investigated the effect of steviol on gastric (HGC-27) and colorectal (Caco-2) cancer cells, it was reported that an increase in the expression of p53 and a decrease in the level of cyclin D occurred. Additionally, the researcher reported that steviol treatment caused G1 arrest in both cell lines [67,68]. Glycyrrhizic acid administration to different gastric cancer cell lines (e.g., MGC-803, BGC-823, SGC-7901) induces cell cycle arrest through the downregulation of G1 phase proteins such as cyclin D1, D2, D3, E1, and E2 [74,77]. In addition, 18β-glycyrrhetinic acid, another metabolite of glycyrrhizin, promoted gastric cancer cell autophagy and induced cell cycle arrest in the G0/G1 phase in a transplanted nude mouse model modulating the miR-328-3p/STAT3 signaling pathway [90]. Similar results were also reported for other cancers, such as cervical cancer [91]. Additional information regarding the observed effect was not reported for other sweeteners, which shows that more collaborative efforts are needed in order to pursue more research in this field. Figure 7 summarizes the effects of sweeteners on cell cycle arrest.

### 4.4. Synthetic Sweeteners and GI Cancers

**Figure 8:**
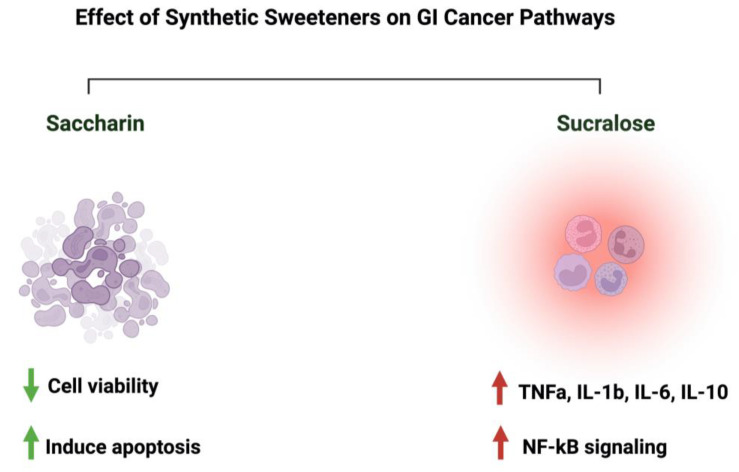
Summary of the influence of synthetic sweeteners on GI cancers. The figure highlights the sweeteners’ positive or negative effects on GI-targeted pathways. Created with BioRender.com (accessed on 15 July 2023).

Due to the controversial results available in the literature regarding the effects of saccharin and sucralose on GI cancer, we decided to discuss them in a separate paragraph. Discussing those two sweeteners raises many questions about their associated risk with gastrointestinal cancers. In an Italian cohort comprising 230 patients with histologically confirmed gastric cancer, after correcting for confounding factors, the researchers reported a lack of adverse effects of saccharin on the risk of developing neoplasms [23]. Additionally, a review paper that discussed 22 cohorts and 46 case–control studies on the effects of sweeteners on different cancers concluded that there was a lack of evidence, but there was a link between saccharin, sucralose, and other sweeteners and cancer risks [92]. Additionally, a study that used the intestinal epithelial cell line Caco-2 to investigate the effects of commonly used sweeteners reported that the administration of saccharin induced apoptosis at a lower concentration (100 uM), while at a higher concentration (1000 uM), it induced cellular death. The same effect was not observed for sucralose [93]. However, other studies reported negative effects of sucralose on colorectal cancer. A murine model administered 1.5 mg/mL of sucralose for six weeks reported a significant increase in the number and size of colorectal tumors. Also, these researchers reported an effect on the gut microbiome and inflammatory markers (TNFa, IL-1b, IL-6, IL-10, and TLR4/Myd88/NF-kB signaling) [94]. The list of studies discussing this effect is growing. However, more efforts from the research community are needed in order to address those differences in a systemic and mechanistic way, as well as to standardize the protocol to be followed and the appropriate dosage used, as it directly affects people’s health through food intake. Figure 8 illustrates the effects of synthetic sweeteners on GI cancers. Table 1 summarizes the available literature on the observed effects of all the sweeteners discussed herein.

## 5. Discussion

### 5.1. Safety of Sweeteners and Challenges in the Field

Recently, the discussion about the safety of one of the commonly used sweeteners in the food industry, “aspartame”, and its possible carcinogenic nature raised more questions about the safety of other sweeteners. Here, and in most of the reported articles, it has been shown that these natural and synthetic sweeteners lack genotoxicity and carcinogenicity and are safe when consumed in moderation [96,97,98,99]. Throughout our research in the literature, most of the utilized concentrations/dosages of the sweeteners did not show adverse negative effects on the model which was used. However, some reports linked the consumption of specific sweeteners to cancer development [94]. Those results show the urgent need to address the field’s main issues. First, protocol standardization, starting from the model used, mode of administration of the sweeteners, duration of the experiment, bioinformatics tools to interpret the results, and estimation of safety measures, is critical to ensure productivity and reproducibility. Second, “recommended dosage” determination, while considering other factors such as geographical location and age, might help us to understand those sweeteners’ consumption rates. Third, guidelines and regulatory process evaluation are crucial to ensure manufacturing safety. Fourth, the possible synergistic effects of sweeteners need further investigation, as these might occur when consuming different products that contain different sweetener types and dosages.

Currently, people are more aware of their health in terms of food and always search for “healthier” and low-caloric options as alternatives while maintaining a sweet taste. The controversy regarding the safety of sweeteners raises another important question: what would be the alternative to using sweeteners? Would we go back to refined sugar, or move toward natural compounds such as flavonoids and phytochemicals? What are the safety and taste estimates of the consumption of those alternatives compared to sweeteners? We have reported the positive effects of flavonoids on GI cancers and the gut microbiome for years. However, more efforts are required in order to evaluate whether they will be a “better” alternative, considering their bioavailability [100,101,102,103]. Additionally, the effect of this “better” alternative on the gut microbiome needs more attention.

Although we encourage more research to be conducted, there are limitations associated with this field. First, the misreporting of participants in terms of the amount/type/quantity of sweeteners consumed might affect the interpretation of the results. Second, selection bias involved in the conducted experiment/tested population would affect the generalizability of the results to the general population. Third, residual confounding shows the urgent need to develop bioinformatics tools that correct for those factors. Fourth, causality concerns are also prominent, along with how to correctly evaluate causality and differentiate it from correlation. Other limitations may include the experimental and interpretational challenges associated with linking specific bacterial species to the metabolism of sweeteners. Addressing those limitations in future studies could help us to improve the research outcomes.

### 5.2. Sweeteners’ Role in Cancer Therapy Development

Based on the results available so far, the consumption of sweeteners in moderation is considered an alternative to consuming refined sugar. Also, using sweeteners is safe and positively influences the development and progression of cancer. What about using these sweeteners to design a therapeutic agent for cancer? A study published in 2014 used isosteviol, a diterpenoid product of the acidic hydrolysis of steviol glycoside, as a potential anti-tumor agent [104]. They synthesized novel isosteviol triazole conjugates using the chemistry method “click”, and they tested the effect of the conjugates on different cancer cell lines such as colorectal cancer, breast cancer, and prostate cancer. They reported that the constructed conjugates showed anti-proliferative activities against cancer cell lines. Although this seems promising, more efforts are required in order to evaluate this method and ensure the stability and safety of using such an agent.

### 5.3. What about Aspartame?

More controversial discussions emerged when the World Health Organization (WHO) announced aspartame as a possible carcinogen. Aspartame is a sweetener used as a replacement for sucrose due to its high sweetness intensity [105]. The effect of aspartame on the gut microbiome has been reported in limited studies. In mice (C57Bl/6) treated with different non-caloric artificial sweeteners, including aspartame, some effect on the gut microbiome abundance and metabolic pathways was reported [16]. Additionally, the fasting glucose concentrations and the abundances of Enterobacteriaceae and *Clostridium leptum* were increased in diet-induced obesity models treated with aspartame for eight weeks [106]. With gastrointestinal cancers being the focus of this review, using aspartame (15 and 30 mM) for HT-29 human colorectal carcinoma proved to have a pro-angiogenic effect [107]. However, consuming artificial sweeteners, including aspartame, was not associated with colorectal or stomach cancers [108]. These data show the urgent need to address those controversial results, putting into perspective the model and the concentration of aspartame used.

## 6. Conclusions

Sweeteners are intense substances used in the food industry as alternatives to table sugar. Debates about the safety and the effect of using those sweeteners on the gut microbiome and the overall health status have gained attention recently. Throughout our study, we reported the relationships between three natural sweeteners (steviol glycoside, glycyrrhizin, neohesperidine dihydrochalcone) and two synthetic sweeteners (saccharin and sucralose) and the gut microbiome. Although relevant to the recent WHO statement, we did not include a detailed analysis of “aspartame” in our analysis, as, to our knowledge, there are limited data on the potential influences of aspartame on the human gut microbiome. We also discussed the effect of either the five sweeteners alone or, if supported by the literature, their metabolites in cancer-related pathways such as apoptosis and cell cycle arrest.

There are differences between countries regarding the various NNS types that are considered safe for human consumption; however, on the other hand, there is no proven linkage to cancer. In this review, we also addressed some of the challenges associated with the field, as well as the efforts required to improve such aspects, such as protocol standardization, systemic evaluation, and guideline regulations. Generally, the gut microbiome’s involvement in sweetener metabolism might be an interesting and promising field for futuristic cancer treatments, primarily when combined with the currently available therapeutics.

## Figures and Tables

**Table 1:** Summary of representative sweeteners/their metabolites and their underlying effects on cancer.

Sweetener Type	Targeted Metabolites/Proteins/Genes/Pathway	Targeted Disease/Tissue	Mechanism of Action	Methods of Testing	Model Used		References
					*In Vivo*	*In Vitro*	
Steviol glycosides	Apoptosis Cellular proliferation	Gastric cancer Colon cancer	- It inhibited mitochondrial apoptotic pathway - Activated p21 and p53 - It increased Bax/Bcl-2 ratio	MTT assay Western blot miRNA analysis Flow cytometry		- HGC-27 cells - Caco-2 cells - HCT-8 cells - HCT 116 cells - MKN-45 cells - MGC-803 cells	[67]
	Cytotoxic Apoptosis	Stomach cancer	- Induced apoptosis cell death - Increased cytotoxicity	MTT assay Apoptotic assays Flow cytometry		- AZ521 cells	[70]
	Apoptosis	Colon cancer	- It decreased cell viability in colorectal cancer cell line	MTT assay Bicinchoninic acid assay	- Wistar rats	- Caco-2 cells	[69]
Neohesperidin dihydrochalcone	Apoptosis Angiogenesis	Colon cancer	- It induced apoptosis and blocked angiogenesis - It altered the gut microbiota	PCR Western blot Luciferase assay Cell survival assay TUNEL assay	- C57BL/6 J - APCmin/+ mice	- HCT116 cells - SW480 cells - CT26 cells	[78]
Glycyrrhizin	Apoptosis	Colon cancer	- Inhibited cellular growth in a dose-dependent manner - It also induced apoptosis through nuclear fragmentation and chromatin condensation	Transmission electron microscopy Apoptotic assay Cell invasion assay Western blot		- SW48 cells	[72]
	Apoptosis Inflammation	Colon cancer	- Treatment with glycyrrhizic acid suppressed the development of early markers of colon cancer - It also suppressed the development of precancerous lesions - Suppressed the immunostaining of NF-Kb and p65	Immunohistochemical staining ELISA Aberrant Crypt Foci (ACF) assay	- Albino rats		[75]
	Inflammation	Colon cancer	- It reduced the plasma level of IL-6 and TNF-a - It significantly reduced the expression of 8- NitroG, 8-OxodG, COX-2, and HMGB1	ELISA Immunohistochemical staining	- ICR mice		[95]
	Apoptosis Inflammation	Colon cancer	- Treatment with glycyrrhizic acid reduced the expression of NF-kB and COX-2 - It enhanced the expression of cleaved caspase 3 - It also reduced the infiltration of mast cells	ELISA Immunohistochemical staining Mast cell staining	- Albino rats		[76]
	Apoptosis Cellular proliferation	Gastric cancer	- Treatment with glycyrrhizic acid downregulated the level of G1 phase-related proteins in a dose- and time-dependent manner - It also upregulated the levels of Bax; cleaved PARP; and pro-caspase-3, -8, -9	CCK-8 assay Apoptotic assay EdU assay Cell cycle assay Western blot		- MGC-803 cells - BGC-823 cells - SGC-7901 cells	[77]
Saccharin	Apoptosis Cell viability	Intestinal epithelium	- At a lower concentration (up to 100 uM), it induced apoptosis, while at a higher concentration (<=1000 uM), it induced cell death - Decreased cell viability and disrupted the intestinal epithelial barrier through binding to the sweet taste receptors	RT-PCR Annexin V assay siRNA and cDNA Transfections ROS assay ELISA	- C57BL/6 mice	- Caco-2 cells	[93]
Sucralose	Inflammation	Colitis-associated colorectal cancer	- Significantly increased the number and size of colorectal tumors - Increased expression of TNFa and TLR4 - Increased the abundance of *Firmicures*, *Clostridium symbiosum*, and *Peptostreptococcus anaerobius* while decreasing the abundance of *Solobacterium moorei* and *Bifidobacteria*	Spectrophotometry qRT-PCR Western blot ELISA	- C57BL/6 mice		[94]

## Data Availability

Not applicable.

## Abbreviations

NNS	non-nutritive sweeteners
GI	gastrointestinal
NHDC	neohesperidin dihydrochalcone
IL-6	interleukin 6
NF-B	nuclear factor kappa-light-chain-enhancer of activated B cells
Bcl-2	B-cell lymphoma 2
TNF	tumor necrosis factor
